# Ground Reaction Force and Moment Estimation through EMG Sensing Using Long Short-Term Memory Network during Posture Coordination

**DOI:** 10.34133/cbsystems.0016

**Published:** 2023-03-27

**Authors:** Sei-ichi Sakamoto, Yonatan Hutabarat, Dai Owaki, Mitsuhiro Hayashibe

**Affiliations:** ^1^ Neuro-Robotics Lab, Graduate School of Biomedical Engineering, Tohoku University, Sendai, Japan.; ^2^ Department of Robotics, Graduate School of Engineering, Tohoku University, Sendai, Japan.

## Abstract

Motion prediction based on kinematic information such as body segment displacement and joint angle has been widely studied. Because motions originate from forces, it is beneficial to estimate dynamic information, such as the ground reaction force (GRF), in addition to kinematic information for advanced motion prediction. In this study, we proposed a method to estimate GRF and ground reaction moment (GRM) from electromyography (EMG) in combination with and without an inertial measurement unit (IMU) sensor using a machine learning technique. A long short-term memory network, which is suitable for processing long time-span data, was constructed with EMG and IMU as input data to estimate GRF during posture control and stepping motion. The results demonstrate that the proposed method can provide the GRF estimation with a root mean square error (RMSE) of 8.22 ± 0.97% (mean ± SE) for the posture control motion and 11.17 ± 2.16% (mean ± SE) for the stepping motion. We could confirm that EMG input is essential especially when we need to predict both GRF and GRM with limited numbers of sensors attached under knees. In addition, we developed a GRF visualization system integrated with ongoing motion in a Unity environment. This system enabled the visualization of the GRF vector in 3-dimensional space and provides predictive motion direction based on the estimated GRF, which can be useful for human motion prediction with portable sensors.

## Introduction

In recent years, along with the development of deep learning, various technologies have been available in our daily life such as voice assistance, image-based medical diagnosis, and language translation. In biomechanics and rehabilitation engineering, deep learning has been employed to develop algorithms for the estimation and prediction of human motion [1–5]. The state of human dynamics during standing or performing certain motions can be reflected using ground reaction force (GRF) data. From this point of view, among the dynamics information, it can be assumed that GRF is quite important for motion prediction. In fact, GRF information is widely used in human motion evaluation for the clinical assessments, such as gait retraining, rehabilitation, and injury prevention. Force plates are often introduced as standard devices along with a motion capture system in biomechanics laboratories. However, despite its importance, GRF acquisition is limited due to cost constraints or the measurable range of the force plate. In addition, it is not available freely for all researchers and clinicians. Hence, the GRF estimation method without a force plate might be important not only for human motion prediction but also for various biomechanical studies.

To the best of our knowledge, most studies on motion prediction use motion capture systems, Kinect, or RGB cameras. In current literature, kinematic information such as human body segment displacements and joint angles are used as inputs for a neural network model. However, few studies have reported on the use of dynamics information for human motion prediction. Because motions are generated originally by forces, it could be difficult to predict force-driven motion, for example, initial movement from a static pose, without dynamics sensing, or in this case, force-related measurement. Therefore, in addition to kinematics information, dynamics information would be beneficial for building better motion prediction systems, especially for actions from static poses. In this study, we propose a method that enables the estimation of dynamics information from wearable sensors using neural networks. For a better motion prediction system and for the further development of biomechanical research, we propose a GRF estimation method using electromyography (EMG) [6–8] in combination with and without an inertial measurement unit (IMU) sensors using recurrent neural networks (RNNs). In particular, long short-term memory (LSTM) has recently been recognized for its high performance in making predictions based on time-series data, including biomedical applications [26,27], and thus, it was employed in this study.

The proposed method uses EMG and IMU signals as inputs to the neural network and provides a GRF estimation as the output. EMG measures the electrical potential generated by muscles when they are neurologically activated, which precedes force generation. Thus, it could be considered that we can get information related to the prediction of subsequent motion from EMG signal of muscle activity. Several studies have been reported on estimating various human dynamics from EMG using neural networks [11–15]. In addition, the EMG-based prediction of arm and hand kinematics for prosthetic and clinical applications has been studied [9,10].

However, these studies are often focused on estimating joint angles and torques or recognizing gesture patterns; thus, there are limited number of studies that have focused on estimating GRF using EMG signals. On the other hand, an IMU sensor that records motion information such as acceleration (ACC) or angular velocity (GYRO) in 3-dimensional (3D) components should be able to improve the GRF estimation performance. To verify the performance under different conditions, we compared the performance of using only EMG and using both EMG and IMU. Some human motions originate purely from muscle activations that are not associated with prior motions, such as the stepping motion (SM) from the static posture. In this case, GRF can be difficult to be estimated without considering the muscle activations. The rest of this paper is organized as follows. The Materials and Methods section explains the experimental setup and protocol, signal processing and data preparation, and our proposed method of using LSTM to estimate GRF and GRM along with the development of the GRF visualization system and the prediction of motion direction based on the estimated GRF. The Results and Discussion section presents the results and discussion. Finally, the Conclusion section concludes the paper and provides directions for future research regarding this topic.

## Materials and Methods

### Experimental devices and procedures

**Fig. 1.**
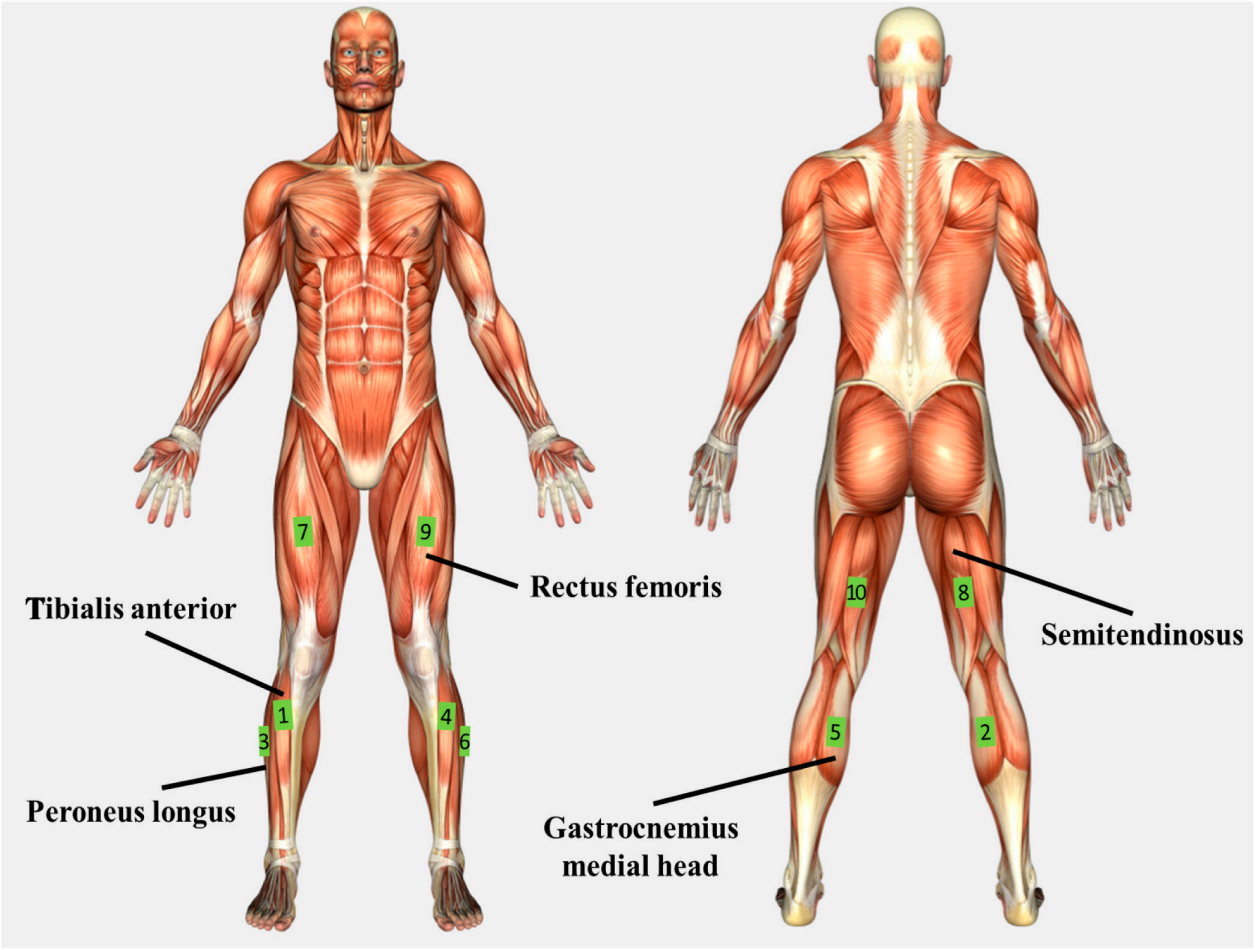
Sensor placement and sensor numbering on its respected muscles.

In this study, we used 10 wireless surface EMG sensors (Delsys Trigno IM Sensor, Delsys Inc., Boston, MA) and 2 force plates (AccuGait, AMTI Inc., Watertown, MA). Because this EMG sensor also has a built-in IMU sensor, both EMG and IMU signals were recorded together using this sensor. The sensors are attached to the tibialis anterior, gastrocnemius medial head, peroneus longus, rectus femoris, and semitendinosus of both legs. Figure 1 shows the location of the muscles where the sensors were attached, while Table 1 shows the sensor numbering. The sampling rates of the EMG and IMU signals were set to 1,111.11 and 148.148 Hz, respectively. First, these 2 signals were filtered, and subsequently, they were downsampled to a sample rate of 74.074 Hz for later to be used as inputs to the neural network. Meanwhile, from the force plates, we measured both GRF and ground reaction moment (GRM) in all 3 axes, that is, Fx, Fy, Fz, Mx, My, and Mz.

**Table 1.** Mapping of sensor position and sensor number.

Muscle name	Right leg	Left leg
Tibialis anterior (TA)	1	4
Gastrocnemius medial head (GAS)	2	5
Peroneus longus (PL)	3	6
Rectus femoris (RF)	7	9
Semitendinosus (ST)	8	10

On the experiment procedures, 2 experiments were designed and conducted with 6 healthy male participants, aged 23 ± 1 years (mean ± SD). The first experiment is posture control motion (PCM), where participants were asked to stand on the force plates, then repeatedly sway their body forward and backward, right and left. They are instructed to maintain their body balance so as to avoid falling. The second experiment involves SM, where participants stood on the force plates, and subsequently stepped their foot left, right, or forward, and move in their self-selected direction repeatedly. Each motion recording session lasted approximately 120 s. The experimental protocol was approved by the ethics committee of Tohoku University under 22A-3. Informed consent for the experimental protocol was obtained from each subject before the experiment was started.

### Signal processing and data preparation

Biosignals are prone to noise exposure. Baseline noise and motion artifacts are examples of the noise found in EMG signals. Thus, it is important to employ filtering process on the EMG signal to remove noise. The 0- to 20-Hz bandwidth of the raw EMG signal contains noise from the motor unit firing, while the bandwidth above 400 Hz has no components derived from muscle contraction [16–18]. Therefore, we used 4th-order butterworth band-pass filter with a pass band of 20 to 450 Hz. The result of this filtering was subsequently forwarded to a 4th-order butterworth low-pass filter with a 3-Hz cutoff frequency to obtain the envelope of the EMG signal. The IMU signal and GRF were also processed by the same low-pass filter to remove noise from those signals [19,20].

After the filtering process, 2 GRF estimation models for PCM and SM, respectively, were trained with different combinations of EMG and IMU signals as inputs, and the recorded GRF and GRM from force plates as the target data. In this study, we prepared 6 input combinations for the GRF and GRM estimation models, as described in Table 2. By changing the combination of the number of sensors and the type of data used as input, we could determined which input and type of data contributed to the design of the LSTM model with the best performance.

**Table 2.** Input combination patterns for the LSTM model investigated in this study.

Pattern no.	EMG no.	IMU no.	Remarks
1	1–10	None	All muscles
2	None	1,4,7,9	IMU on thighs and shanks
3	1–10	1,4,7,9	All muscles and IMU on thighs and shanks
4	1–6	None	Muscles (TA, GAS, PL)
5	None	1,4	IMU on shanks
6	1–6	1,4	Muscles (TA, GAS, PL) and IMU on shanks

### Model building and performance evaluation

GRF and GRM were estimated from EMG and IMU signals using a neural network. Because EMG and IMU signals are time dependent, it is necessary to use a neural network model that is capable of processing time-series data. The standard RNN model is one of the easiest choices, but it can experience challenges in learning long time-span data. As the time lag between relevant input events and output events increases, it becomes difficult to learn these dependencies because of gradient vanishing and explosion during back propagation [21]. Therefore, a standard RNN is not appropriate for situations in which long time steps between input and output events exist, such as EMG signals and force generation. To address this time lag problem, we employed an LSTM model for GRF and GRM estimation. LSTM follows an RNN architecture with added features called memory cells (cell state) and gates that are not found in the standard RNN model [21]. The memory cells store and transfer past information, which enables the prediction of long-time dependency data. The gates are categorized into 4 types, which are called forget gate (*f_t_*), generation gate (*g_t_*), input gate (*i_t_*), and output gate (*o_t_*). Each gate modifies the information stored in the memory cell or determines the output of the LSTM layer. The computations carried out by these gates are expressed using Eqs. 1 to 4, where *f_t_* gates are functioned to delete unnecessary information from the memory cell, and *g_t_* serves to generate new information and add it to the memory cell.

The computation of cell state (*c_t_*) and output of LSTM model (hidden state) at time *t*, *h_t_*, are determined by Eqs. 5 and 6, respectively.

$$f_{t}=\sigma \left(x_{t}W_{x}^{\left(f\right)}+h_{t-1}W_{h}^{\left(f\right)}+b^{\left(f\right)}\right)$$ (1)

$$g_{t}=\sigma \left(x_{t}W_{x}^{\left(g\right)}+h_{t-1}W_{h}^{\left(g\right)}+b^{\left(g\right)}\right)$$ (2)

$$i_{t}=\sigma \left(x_{t}W_{x}^{\left(i\right)}+h_{t-1}W_{h}^{\left(i\right)}+b^{\left(i\right)}\right)$$ (3)

$$o_{t}=\sigma \left(x_{t}W_{x}^{\left(o\right)}+h_{t-1}W_{h}^{\left(o\right)}+b^{\left(o\right)}\right)$$ (4)

$$c_{t}=f_{t}⊙c_{t-1}+g_{t}⊙i_{t}$$ (5)

$$h_{t}=o_{t}⊙\text{tan}h\left(c_{t}\right)$$ (6)

where *σ*(·) is the sigmoid function, *x* is the input vector, *h* is the output vector, *W* is the weight matrix for *x* and *h*, and *b* is the bias at each gate computation. Each superscript means gate type, and each subscript means time step.

**Fig. 2.**
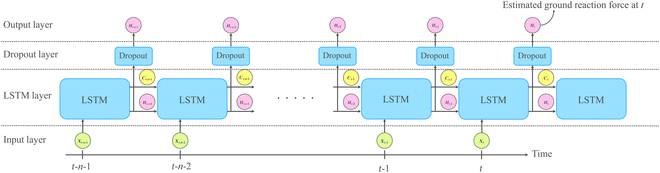
Temporal flowchart of ground reaction force estimation model with dropout layer configuration.

These models were then trained using the architecture of the LSTM model as follows: A single LSTM layer with 64 hidden units and a 50% dropout probability was employed to predict GRF and GRM from different input combination patterns, as mentioned in the Signal processing and data preparation section, with a unit time step. This configuration is well suited for estimating time-series data as found in [22]. A dropout layer is introduced to prevent overfitting problem during the learning phase. We used 50% of the dataset as the training data and the other 50% as the testing data. Meanwhile, the batch size was set to 64, and the learning rate was set to 0.001. For the optimization algorithm, we employed the Adam optimizer [23]. Using this LSTM architecture, we proposed a GRF estimation model as depicted in Fig. 2. After the training phases, both trained models of PCM and SM were evaluated using test data that were not used for the training phase. The root mean square error (RMSE) was calculated as the estimation performance index.

### Development of GRF visualization system

In this study, we also developed a GRF visualization system by integrating the proposed GRF estimation method and Unity used for game development. The estimated and measured GRF were sent to Unity using Transmission Control Protocol (TCP) communication, and the GRF vector calculated by the LSTM model was visualized in 3D space created in Unity. To visualize the participant motion with GRF, the Neuron Pro (Noitom Ltd., Miami, Florida) motion capture system was used and its data were simultaneously streamed to Unity. This motion capture system enables the automatic creation of a human skeleton model by attaching sensors to several locations on the participants’ body. Overall, the tracking of the skeleton data, measured GRF from the force plate for validation, and estimated GRF from the LSTM model were sent to Unity to be visualized. The developed system enabled us to observe the estimated and measured GRF vectors together with the skeletal motion of the user in the PCM experiment. Meanwhile, in the SM experiment, we added a predictive function of motion, which indicates the direction where participants are moving to, as shown by the green arrow at the top of the skeleton model. The arrow direction is determined based on the force components in the estimated GRF data. As forces precede the motion generation, it can lead to a faster prediction as compared to the prediction based on positions or derivatives.

## Results and Discussion

### GRF estimation for PCM

**Fig. 3.**
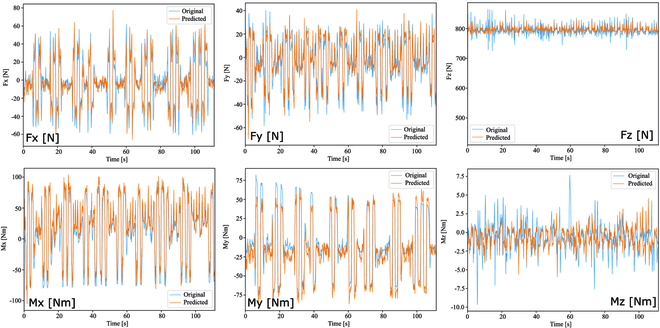
Graphical comparison between estimation (yellow) and measurement (blue) of GRF and GRM on PCM.

Figure 3 shows the results of GRF and GRM estimation using the proposed LSTM model with input pattern no. 6 for the PCM experiment setting, where the yellow lines represent the estimation results, and the blue lines represent the measurement from force plates. The results demonstrate that the learned LSTM model can successfully follow the posture control movement pattern performed by the subjects as reflected by the GRF patterns with an average estimation error of less than 10% as compared to the ground truth from the force plates. Table 3 summarizes the results of the GRF estimation in the PCM experiment. The values in the table represent the RMSE [%] between the estimated and measured GRFs, regarding the translational force and the rotational moment in terms of the Cartesian coordinates of the force plate. We also provided the standard error (SE) metric to measure the distribution of data from the mean. In the cases of both above and below knee sensing, we observed that for input pattern no. 1 where all EMG sensors were used as input data without using IMU sensors, the GRF estimation performance in terms of RMSE is 9.94%. In the case of input pattern no. 2 where we used 4 IMU sensors on both thighs and shanks, the RMSE decreased by an average of 2.11% as compared to input pattern no. 1. Moreover, in the case of input pattern no. 3, we observed a slight increase in the RMSE by an average of 0.06% as compared to input pattern no. 2.

**Table 3.** GRF and GRM estimation error on posture control motion.

Input	Fx [N]	Fy [N]	Fz [N]	Average ± SE [N]	Mx [Nm]	My [Nm]	Mz [Nm]	Average ± SE [Nm]
1	11.2%	11.3%	7.33%	9.94% ± 1.31%	13.2%	16.3%	11.3%	13.6% ± 1.46%
2	7.83%	9.00%	6.67%	7.83% ± 0.67%	9.50%	7.33%	9.83%	8.89% ± 0.78%
3	7.67%	9.17%	6.83%	7.89% ± 0.68%	7.83%	7.17%	9.33%	8.11% ± 0.64%
4	11.0%	11.8%	7.33%	10.04% ± 1.38%	13.5%	18.2%	11.3%	14.33% ± 2.03%
5	8.17%	11.3%	6.67%	8.71% ± 1.36%	16.8%	7.83%	10.0%	11.54% ± 2.70%
6	8.00%	10.0%	6.67%	8.22% ± 0.97%	10.7%	7.67%	9.67%	9.35% ± 0.89%

On the other hand, for input pattern nos. 4 to 6 with sensors below the knees, the trend of the RMSE results was similar to input pattern nos. 1 to 3, in terms of the contribution relationship of EMG and IMU to the estimated GRF. It can be observed from Table 3 that for input pattern no. 4, where 6 EMG sensors below the knee were used without using IMU sensors, the RMSE increased by an average of 0.1% compared to when we used 10 EMG sensors on input pattern no. 1. This result may indicate that upper leg muscle activities may not contribute substantially to estimating GRF in posture control movement activity. Input pattern no. 5 with 2 IMU sensors on both shanks showed an average increase of 0.88% in RMSE as compared to input pattern no. 2 where we used 4 IMU sensors on both thighs and shanks, and showed an average decrease of 1.33% in RMSE as compared to input pattern no. 5 where only below knee EMG data were used. Finally, for input pattern no. 6, a combination of input pattern nos.4 and 5, we observed an average increase of 0.33% in the RMSE as compared to input pattern no. 3, and showed an average decrease of 0.49% in the RMSE.

It should be noted that only EMG and IMU sensors below the knees could estimate the measured GRFs without a significant increase in RMSE. This fact may also be related to the issue of Ankle Strategy for postural control. This experiment was performed using a self-controlled balance task, and the ankle joint was principally employed.

### GRF estimation for SM

**Fig. 4.**
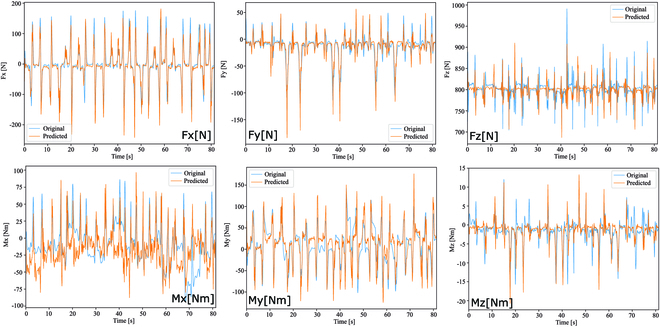
Graphical comparison between estimation (yellow) and measurement (blue) of GRF and GRM on SM.

Figure 4 shows the results of GRF and GRM estimation using the proposed LSTM model with input pattern no. 6 for the SM experiment setting, where the yellow lines represent the estimation results and blue lines represent the measurement from force plates. The results demonstrate that the learned LSTM model can successfully follow the SM pattern performed by the subjects as reflected by the GRF patterns with an average estimation error of less than 12% as compared to the ground truth from the force plates, and a 2% higher on average estimation error as compared to the result from the PCM experiment. Table 4 summarizes the results of GRF estimation in the SM experiment. In the cases of both above and below knee sensing, we observed that for input pattern no. 1 where 10 EMG sensors were used as input data without using IMU sensors, the GRF estimation performance in terms of RMSE is 13%. For input pattern no. 2 where we used 4 IMU sensors on both thighs and shanks, the RMSE decreased by an average of 1.57% as compared to the result from input pattern no. 1. Further, in the case of input pattern no. 3, a combination of input pattern nos.1 and 2, we observed a decrease in the RMSE by an average of 0.92% as compared to input pattern no. 2.

**Table 4.** GRF and GRM estimation error on stepping motion.

Input	Fx [N]	Fy [N]	Fz [N]	Average ± SE [N]	Mx [Nm]	My [Nm]	Mz [Nm]	Average ± SE [Nm]
1	11.0%	11.5%	16.5%	13% ± 1.76%	12.8%	14.7%	10.3%	12.6% ± 1.27%
2	7.3%	11.3%	15.7%	11.43% ± 2.43%	12.0%	8.83%	9.33%	10.05% ± 1.20%
3	7.17%	9.17%	15.2%	10.51% ± 2.41%	11.0%	8.83%	8.83%	9.55% ± 0.72%
4	11.0%	11.7%	17.0%	13.23% ± 1.89%	13.3%	15.0%	10.3%	12.87% ± 1.37%
5	8.50%	11.3%	16.0%	11.93% ± 2.19%	16.0%	10.3%	10.0%	12.1% ± 1.95%
6	8.00%	10.2%	15.3%	11.17% ± 2.16%	12.3%	10.2%	9.50%	10.67% ± 1.05%

On the other hand, for input pattern nos. 4 to 6 with sensors below the knees, the trend of the RMSE results was similar to nos. 1 to 3, in terms of the contribution relationship of EMG and IMU to the estimated GRF. It can be observed from Table 4 that for input pattern no. 4 where 6 EMG sensors were used below the knee without using IMU sensors, the RMSE increased by an average of 0.23% as compared to when we used 10 EMG sensors in input pattern no. 1. The RMSE difference of this result is more than twice than that of the PCM experiment, which could indicate that upper leg muscle activities may contribute more to estimating GRF in SM. Input pattern no. 5 with 2 IMU sensors on both shanks showed an average increase of 0.5% in RMSE as compared to input pattern no. 2 where we used 4 IMU sensors on both thighs and shanks, and showed an average decrease of 1.3% in RMSE as compared to input pattern no. 5 where only below knee EMG data were used. Finally, for input pattern no. 6, a combination of input pattern nos. 4 and 5, we observed an average increase of 0.66% in RMSE as compared to input pattern no. 3, and showed an average decrease of 0.76% in RMSE as compared to input pattern no. 5. Similar to the PCM case, when we used below knee sensors only, the accuracy did not decrease significantly. Therefore, we can infer that the ankle joint muscles are predominantly employed for the stepping action.

### GRF visualization and application for motion prediction during SM

**Fig. 5.**
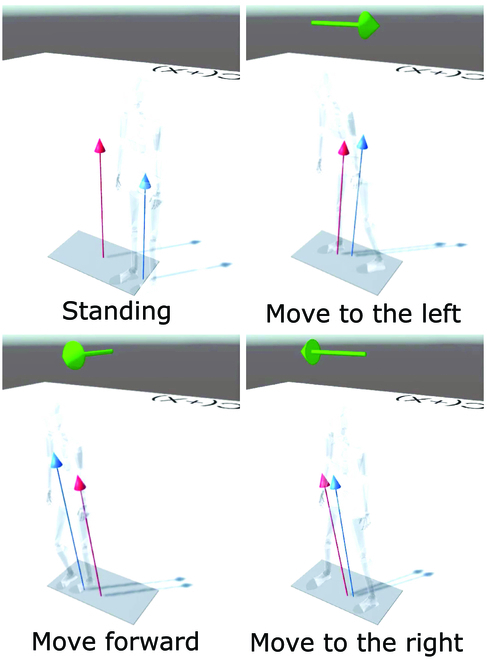
Visualization of GRF vector with human skeleton model, where the red arrow is the measurement from force plate, the blue arrow is the estimation from the LSTM model, and the green arrow is the prediction of stepping motion direction.

In the SM experiment, we visualized the estimated and measured GRF vectors using a human skeleton model as shown in Fig. 5. The system function can also be confirmed using the associated video submitted with this paper. The blue arrow represents the estimated GRF vector, and the red arrow represents the measured GRF vector. We observed that the direction of 2 arrows corresponds well for each directional motion (moving left, right, and forward). The proposed method enabled us to obtain the GRF level with an estimation accuracy of approximately 10%. Force level prediction can be well used for motion prediction, since the center of mass motion is always initiated from the GRF change. As we can know the GRF change, we can apply simple support vector machine decoding to predict the motion direction in the early phases of an action. Figure 5 represents the results of the motion prediction indicated by the green arrow. We can observe that the participant stepping direction is well detected using the dynamics information, which is the estimated GRF.

### Discussion

This study aimed to estimate GRF and GRM in a non-motion capture environment. Even by using the IMU unit, an accurate measurement of acceleration could not be extracted; thus, using only dynamic equations may not produce an accurate estimate of GRF. Therefore, we proposed a method to estimate GRF and GRM by using the LSTM model that was designed to map the available input data, which in this case is the EMG in combination with and without IMU, to the target data of GRF and GRM. We have verified the estimation performance with different input combinations over EMG and IMU, above the knee and below knee sensing. The results indicated that the trained LSTM model can estimate all 3 axes of GRF with an accuracy of 89.96% to 92.17% even only with EMG sensing without an IMU in the PCM experiment. We found that input pattern no. 2 performed the best among the input patterns with an average RMSE of 7.83%, followed closely by input pattern no. 3 with an average RMSE of 7.89%. If we also accounted for the GRM estimation result, we found that the best estimation results were obtained when we used both the EMG and IMU sensors (input pattern no. 3). Meanwhile, for the SM experiment, we found that the trained LSTM model could estimate all 3 axes of GRF with an accuracy of 86.77% to 89.49% depending on the input pattern. We also found that input pattern no. 3, where we used 10 EMG and 4 IMU data as inputs, performed the best among the other input patterns in estimating both GRF and GRM.

Although we reduced the number of sensors, that is, we used sensors only below the knees, the RMSE did not decrease more than 2.25%; hence, the decrease in RMSE was not significant. For the case with reduced IMU sensors (from no. 2 to no. 5), the RMSE of *M_x_* increased compared with only the EMG cases (from no. 1 to no. 4). In other words, it would be difficult to estimate *M*_x_ accurately using only the IMU sensor attached under the knees. When participants swayed their body forward and backward, the body segments above the knees are more accelerated, suggesting that the IMU sensors above the knees contribute to estimating the *M_x_* components of the force plate. On the other hand, participants continuously used muscles below the knees during both PCM and SM, resulting in a smaller increase in *M_x_* RMSE for only EMG cases.

Regarding *M_x_*, the estimation error increased by 7.3% when the IMU sensors were reduced below the knee for posture control. A 4.0% error increase was also observed for the SM. This error increase could be compensated by the usage of the EMG sensor, and then the IMU and EMG input cases could manage the *M_x_* estimate. If we observed the difference in the estimation error between no. 5 and no. 6 row in the table, it is obvious to notice that a large gap exists only regarding for *M_x_*. The gap was 6.1% difference for posture control and 3.7% difference for SM. This implies that EMG information is essential for *M_x_*. It also implies that this moment can be generated directly by muscle activation without being associated with body segment motions. Therefore, the results presented here strongly support that GRF and GRM estimation with the combination of EMG and IMU sensors could contribute to enhancing estimation over various types of human motor control, as forward and backward motion of the body is often used in daily life.

## Conclusion

In this study, we investigated the GRF and GRM estimation for PCM and SM from EMG in combination with and without the IMU sensors using the LSTM network architecture. The results demonstrated that GRF can be estimated with an RMSE of 8.22 ± 0.97% (mean ± SE) for the PCM and 11.17 ± 2.16% (mean ± SE) for the SM, based only on sensors attached under the knees as inputs. We confirmed that EMG input is essential, particularly when we need to predict both GRF and GRM with a limited number of sensors attached under the knees. In addition, we successfully developed a GRF visualization system that incorporates the proposed GRF estimation method and motion capture input in a Unity environment. This system enabled us to observe the GRF vector along with human skeleton model. Moreover, we implemented a predictive function to infer the motion direction from the force components of the estimated GRF during the stepping action of a subject, which can be normally difficult to predict as stepping can occur from the static pose.

As mentioned earlier in the Introduction, human motion originates from forces. To improve motion prediction, it is important to use dynamics information in addition to kinematics information. In terms of interaction with the surrounding environment, it can be assumed that the GRF information is quite important for generating human motion. Our proposed method enables the GRF estimation in a cost-efficient manner. In addition, the sensor is portable and can visualize the GRF vector with a human skeleton model. In addition, the current study adopted LSTM as it is recently recognized for its high performance to making predictions based on time-series data. The latest new computational methods, such as the Transformer, may further improve the current results for future studies.

We believe that this method can also be beneficial for other related biomechanical research by obtaining a GRF estimate in a scenario where we can not use the force place. In a future work, we plan to extend this method to be integrated with motion performance, motion discrimination information [24], and time-scale considered motion synergy information [25,26] for rehabilitation purposes.

## Data Availability

The data that support the findings of this study are available from the corresponding author upon reasonable request.

## Supplementary Materials

Supplementary 1 Movie S1. Ground reaction force and moment estimation with online visualization.
